# Supplementary material for: A Set of Cell Lines Derived from a Genetic Murine Glioblastoma Model Recapitulates Molecular and Morphological Characteristics of Human Tumors
Source: Cancers (Basel). 2021 Jan 10;13(2):230. doi: 10.3390/cancers13020230 (PMC7827614; doi:10.3390/cancers13020230)
Supplement: Supplementary file 1 [file cancers-13-00230-s001.zip › Supplementary material/cancers-1033657-supplementary_BC.docx]

Supplementary Materials: A Set of Cell Lines Derived from a Genetic Murine Glioblastoma Model Recapitulates Molecular and Morphological Characteristics of Human Tumors

Barbara Costa, Michael N.C. Fletcher, Pavle Boskovic, Ekaterina L. Ivanova, Tanja Eisemann,
Sabrina Lohr, Lukas Bunse, Martin Löwer, Stefanie Burchard, Andrey Korshunov, Nadia Coltella, Melania Cusimano, Luigi Naldini, Hai-Kun Liu, Michael Platten,
Bernhard Radlwimmer, Peter Angel and Heike Peterziel


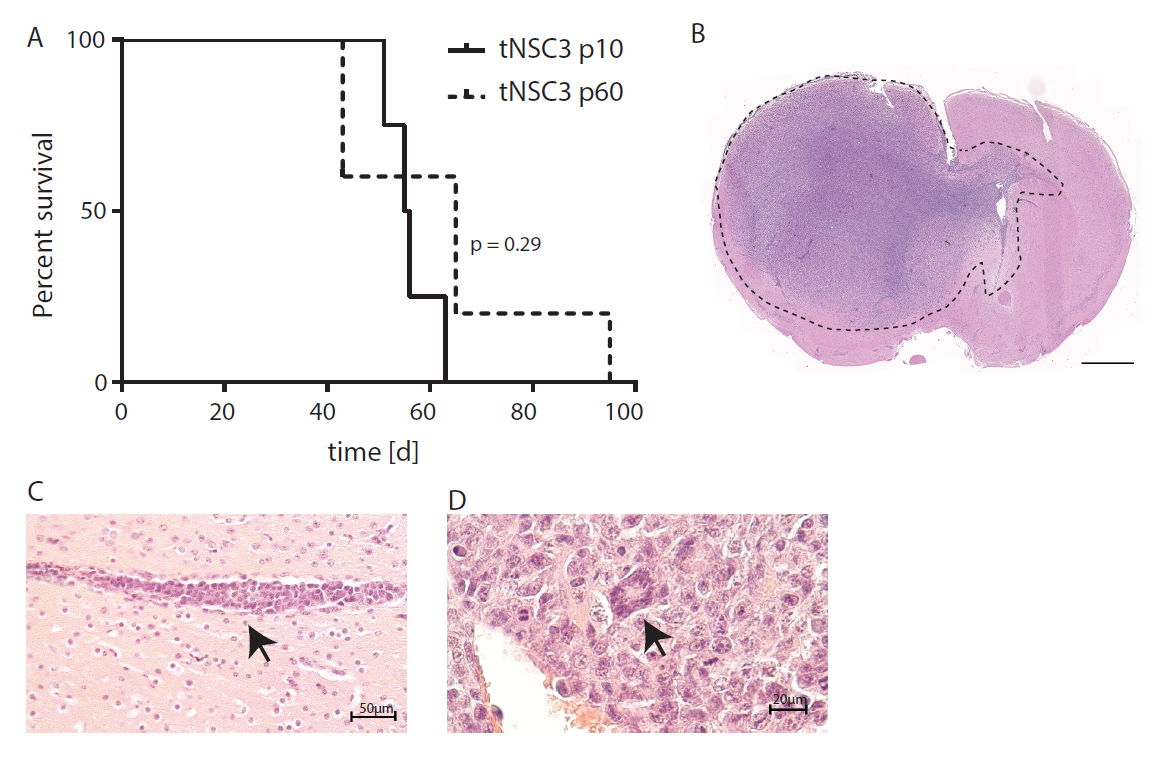


**Figure S1. (A)** Kaplan-Meier survival curve of mice transplanted with tNSC3 cells at early (p10) and late passages (p60). Time = days. Statistical analysis: Mantel-Cox test; (**B)** Representative picture of a tNSC3 orthotopic glioma derived from cells at late passage (p60). Section was stained with hematoxylin and eosin (H&E). Scale bar = 1000 µm. Tumor area is delineated by a dotted line. (**C,D**) Histopathological features of orthotopic tNSC3 late passage gliomas. Sections were stained with hematoxylin and eosin (H&E). Arrow in C denotes areas of perivascular growth; arrow in D indicates a mitotic figure.

**Figure S2.** (**A**) Schematic representation of RNA sequencing reads mapping on p53 and Pten. Blue boxes represent exons. The red rectangles highlight the lack of coverage in exons 2–10 for p53 and exon 5 for Pten in the tNSC and GBM cell lines. These exons are deleted in the DKO genetic mouse model following tamoxifen-induced cre-mediated recombination. (**B**) Gene Set Enrichment Analysis plot for the mSigDB Hallmark pathway “p53-regulated genes” in tNSCs (left panel) and GBMs (right panel) cell lines, compared to ctrlNSCs. (**C**) Table showing RNA transcript reads and genotype for *Idh1* and *Idh2* mutation-specific codons that correspond to the hotspots commonly mutated in human glioma samples. (**D)** Gene Set Enrichment Analysis plot for mSigDB “Markey_RB1_Acute_LOF_UP” in tNSCs cell lines versus ctrlNSCs. **(E)** Gene expression values (voom log-cpm) of an activated NSC gene signature (n=164 genes) in each sample. Each point represents a single gene. Boxplots show the median (central line), first and third quartiles (box) and 1.5x the IQR (whiskers).


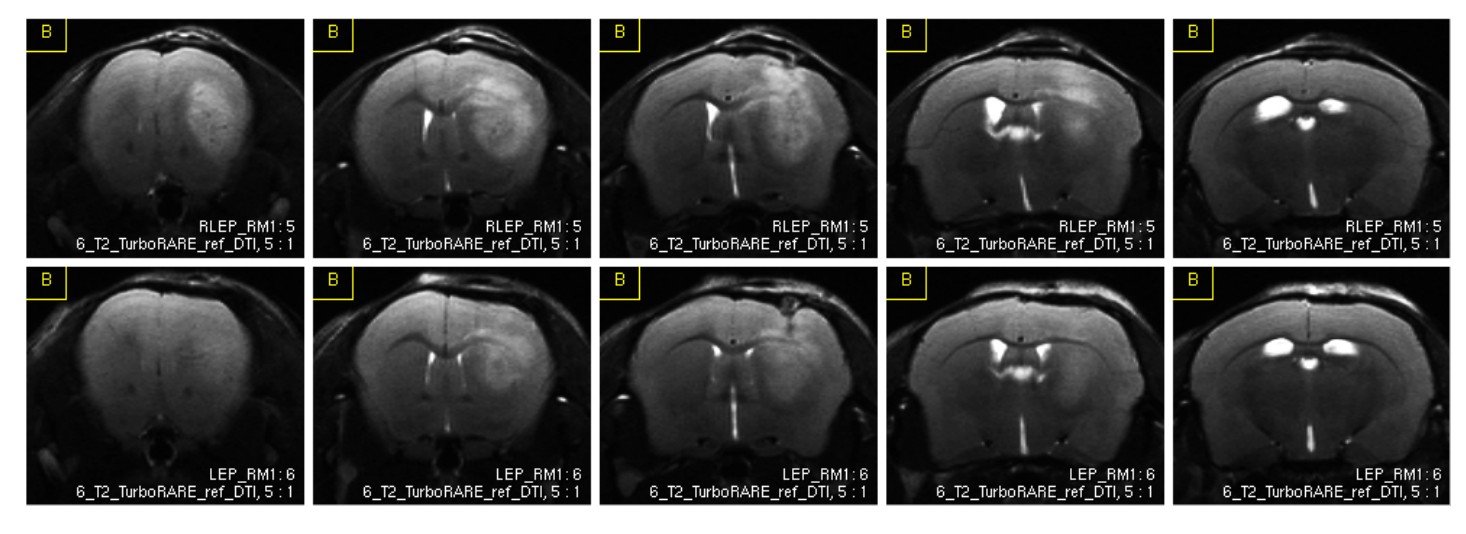


**Figure S3.** T2-weighted magnetic resonance brain imaging (MRI) series of two representative mice 41 days after mGB2 cell transplantation. The hyperintense signals in the right hemispheres reflect the tumors.

**Figure S4.** Immunofluorescence staining for CD31 and CD34 to detect blood vessels within tumor areas (upper pictures) and in the surrounding brain parenchyma (lower pictures) of an mGB2 glioma. Cellular nuclei are stained with DAPI and pseudocolored in blue.

**Figure S5.** Ki67 expression in orthotopic mGB0, mGB1 and mGB2 tumors detected by immunohistochemistry. The sections were counterstained with hematoxylin. N indicates a necrotic area. H indicates tumor areas with hemorrhages.
